# Supplementary material for: Reconstructing historical climate fields with deep learning
Source: Sci Adv. 2025 Apr 2;11(14):eadp0558. doi: 10.1126/sciadv.adp0558 (PMC11963980; doi:10.1126/sciadv.adp0558)
Supplement: Supplementary file 1 — Figs. S1 to S13 References [file sciadv.adp0558_sm.pdf]

Science Advances
AAAS

Supplementary Materials for

Reconstructing historical climate fields with deep learning

Nils Bochow *et al.*

Corresponding author: Nils Bochow, nils.bochow@uit.no

Sci. Adv. **11**, eadp0558 (2025)
DOI: 10.1126/sciadv.adp0558

This PDF file includes:

Figs. S1 to S13
References

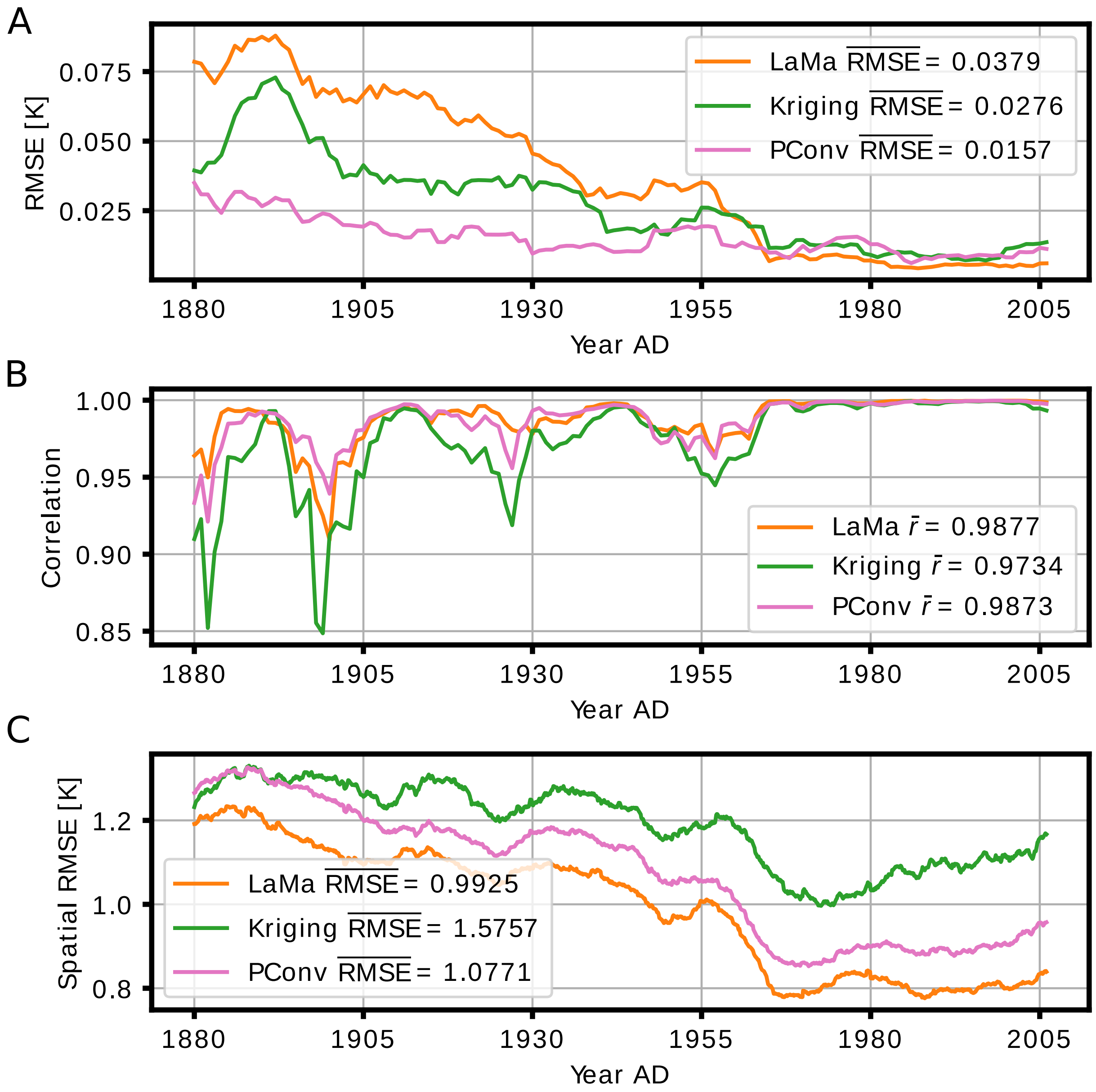

Figure S1: **Comparison with reconstruction method via PConv and kriging for held-out CMIP5 member. (A)** Root-mean-squared error between infilled yearly time series for LaMa, kriging and PConv (*15*) and the held out CMIP5 member (ground truth) in a rolling window with size $w = 10$ years (1870-2005 AD). The mean RMSEs over the whole time period are denoted in the legend. **(B)** Same as **(A)** but for correlation between yearly temperature time series. **(C)** Weighted spatial root-mean-squared error of monthly temperature fields in a rolling window with window size $w = 10$ years.

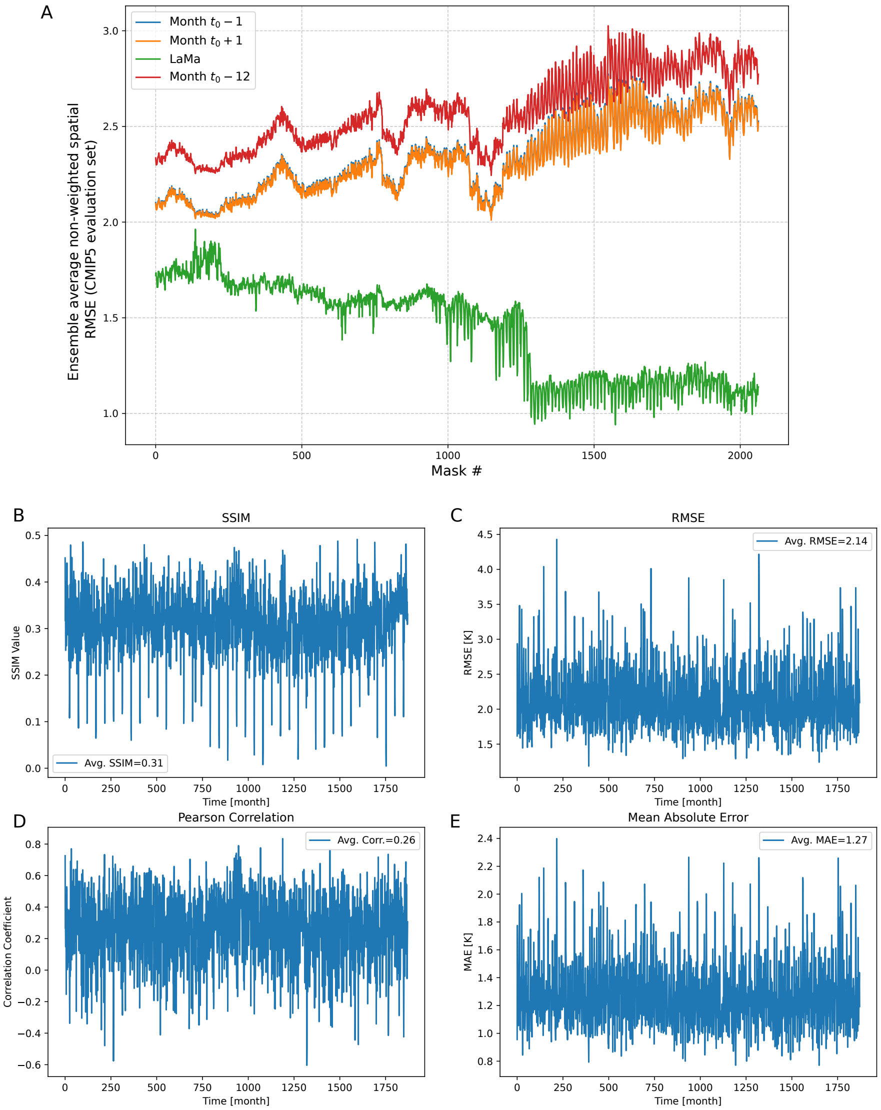

Figure S2: **Analysis of similarity of CMIP5 evaluation data set. (A)** Ensemble mean of the spatial root-mean-squared error (non-weighted) of the reconstruction via LaMa and the naive approach of filling in the missing grid cells with the previous month (Month $t_0 - 1$), the following month (Month $t_0 + 1$) and the same month of the previous year (Month $t_0 - 12$) on the CMIP5 evaluation set. We calculate the spatial RMSE of each ensemble member (2251) average for each mask. The RMSE of the LaMa reconstruction is generally lower than of the naive approaches. **(B)** Structural similarity index measure (SSIM) between consecutive months of one model of the CMIP5 ensemble. A SSIM of 1 indicates perfect similarity between two images, while 0 corresponds to no similarity (*62*). The average metric is depicted in the legend. **(C,D,E)** Same as **B** but for the RMSE, Pearson correlation and MSE, respectively. Generally, the similarity between consecutive months is low.

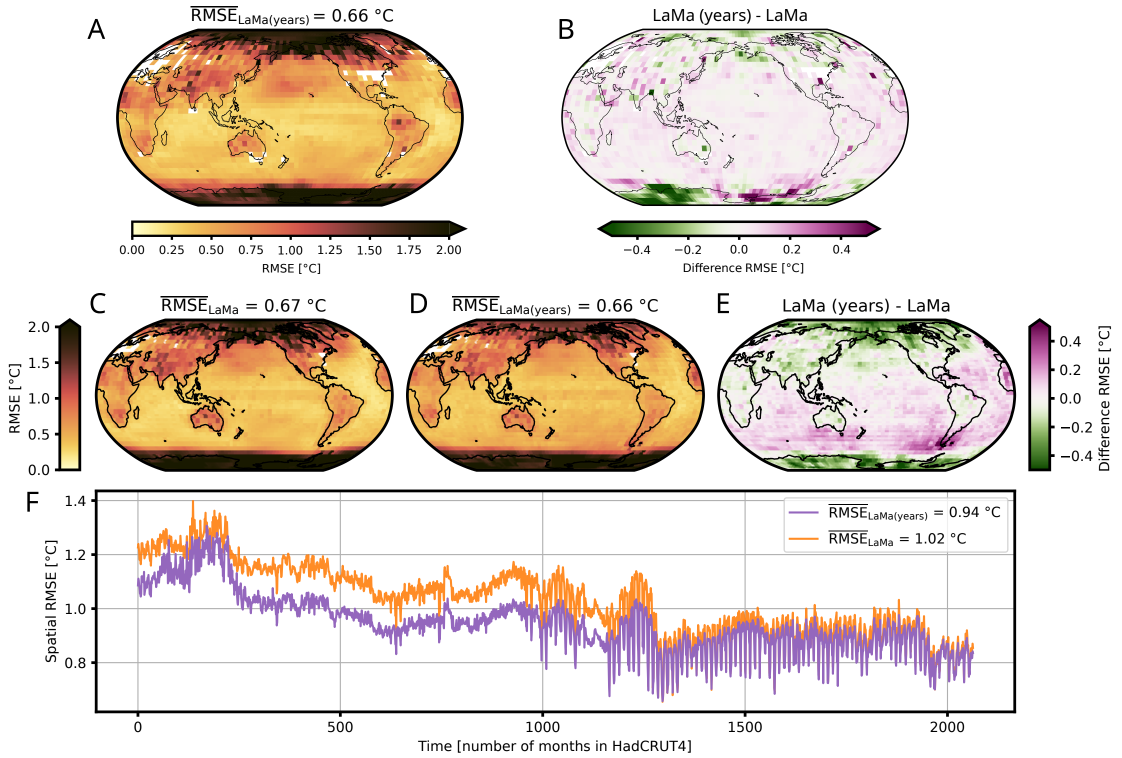

Figure S3: **Error metrics for LaMa trained on alternative train-validation-test split.** **(A)** Site-wise RSME for LaMa trained by withholding random years instead of every 9th month (named LaMa (years)) on the 145th CMIP5 ensemble member. **(B)** Difference in site-wise RMSE between LaMa (years) and LaMa shown in the main text on the 145th CMIP5 ensemble member from 1870-2005. Green areas denote regions where LaMa (years) has a lower RMSE than LaMa. **(C)** Site-wise RMSE of LaMa on the 2251 withheld months (test set) as shown in the main text. **(D)** Site-wise RMSE of LaMa (years) on the alternative test set (3456 monthly fields). **(E)** Difference in site-wise RMSE between the two models. Green areas denote regions where LaMa (years) has a lower RMSE than LaMa. Note that the two test sets are not the same. **(F)** Spatial RMSE for both methods and all HadCRUT4 masks, which are ordered in time; note that generally the size of the masks in terms of the number of missing data declines over time. LaMa (years) shows a lower spatial RMSE for almost all masks.

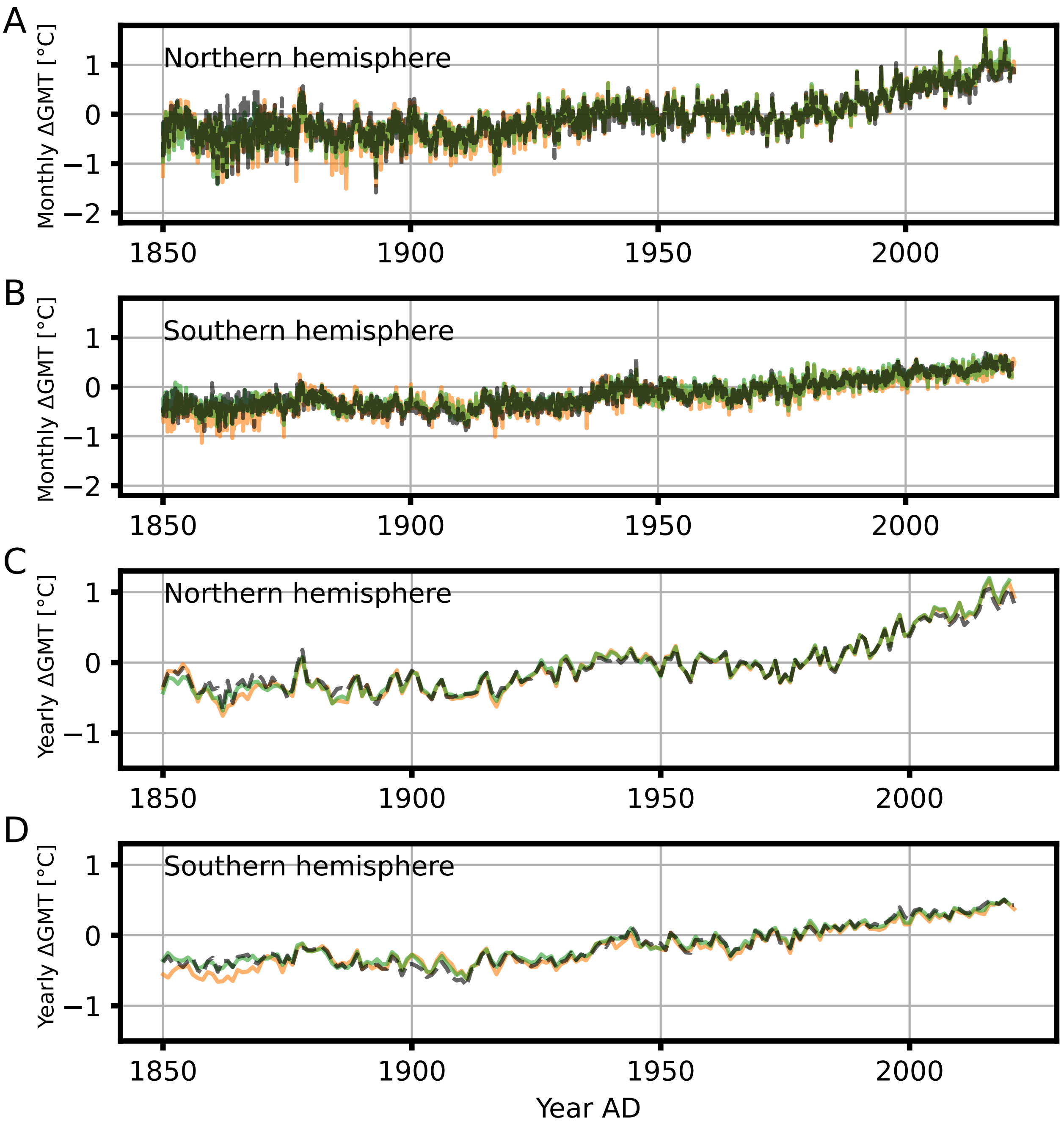

Figure S4: **Reconstructed HadCRUT4 global mean temperature time series for Northern and Southern Hemisphere. (A)** Monthly HadCRUT4 timeseries from 1850 to 2022 for the Northern Hemisphere relative to the period 1960-1990. The reconstructions from LaMa and kriging as well as the mean of the masked HadCRUT4 records are shown. The dashed black curve is the spatially averaged GMT derived from the incomplete HadCRUT4 observations. **(B)** Same as **A** but for the Southern Hemisphere. **(C)** Same as **A** but for the yearly averaged GMT. Additionally, we show the reconstruction based on the PConv method. **(D)** Same as **C** but for the Southern Hemisphere.

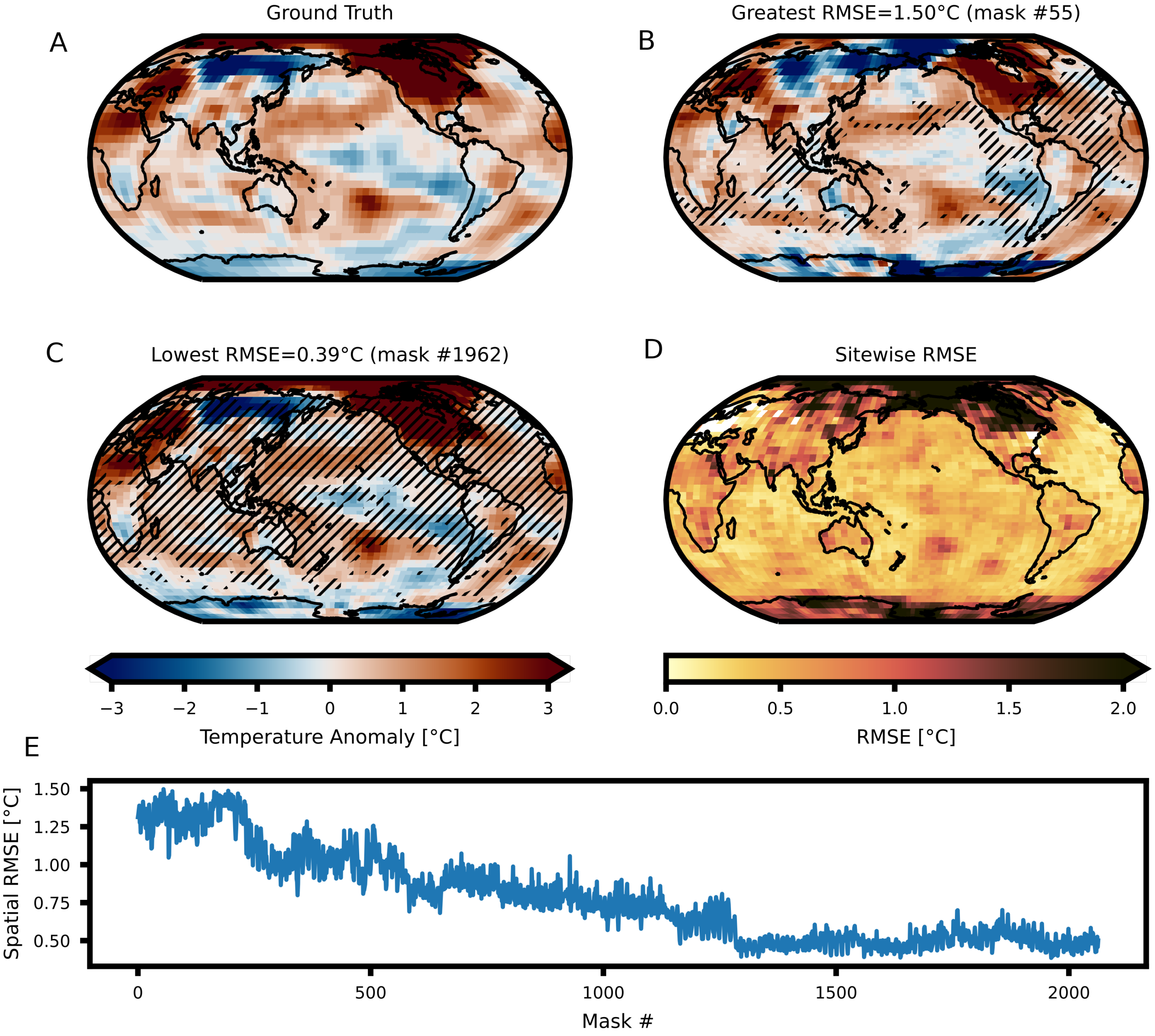

Figure S5: **Reconstructed HadCRUT5 temperature field for January 2021 with HadCRUT4 derived masks.** **(A)** Original HadCRUT5 temperature anomaly for January 2021 (ground truth). **(B)** Reconstructed temperature anomalies with LaMa for the month with the highest spatially weighted spatial root-mean squared error. The hatched area denotes the grid cells that are unmasked, i.e. the information fed into LaMa. **(C)** Same as **B** but for the month with the lowest RMSE. **(D)** Mean site-wise RMSE for the all applied masks. White areas denote grid cells with information available throughout all masks. **(E)** Time series of the spatially weighted spatial RMSE for all applied masks.

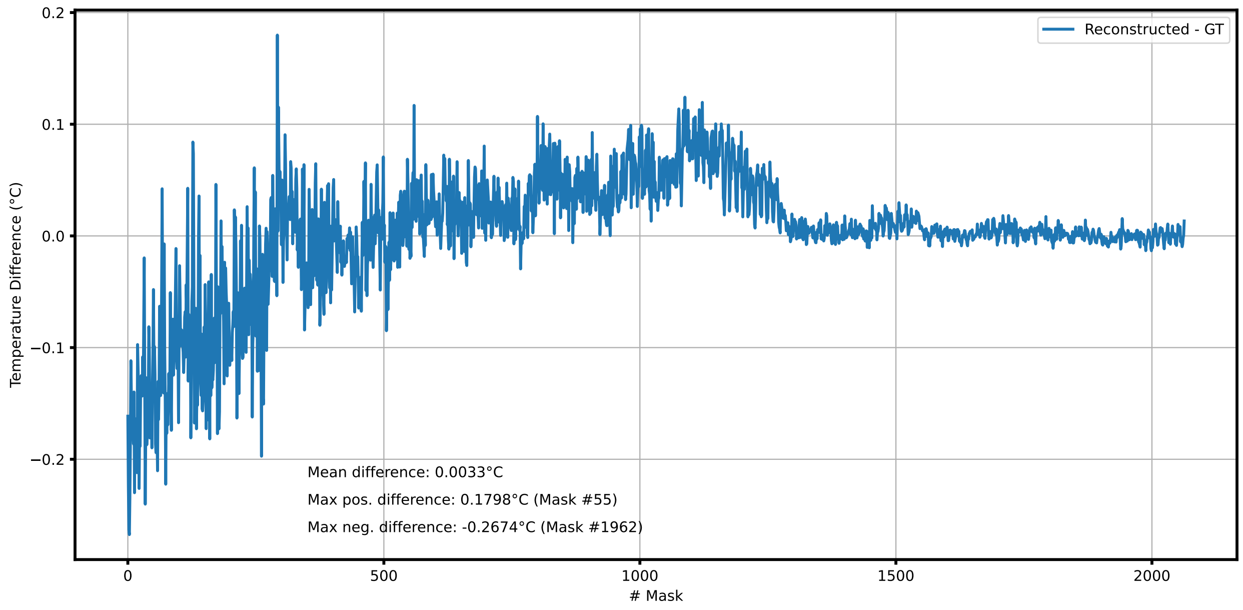

Figure S6: **Difference in GMT between HadCRUT5 temperature field for January 2021 and reconstruction.** Temperature difference between ground truth GMT for January 2021 derived from the HadCRUT5 data set and the reconstructed GMT. We apply all HadCRUT4 derived masks and reconstruct the artificially masked HadCRUT5 temperature field.

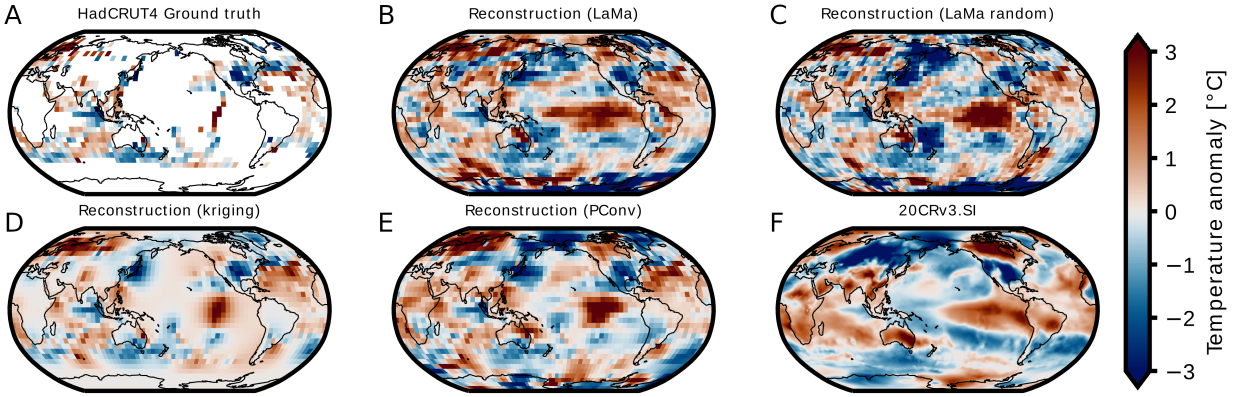

Figure S7: **Reconstructed HadCRUT4 temperature field for November 1877. (A)** Original HadCRUT4 temperature anomaly records. This also corresponds to the input into the trained model. **(B)** Reconstructed temperature anomalies with LaMa. The strong El Niño is clearly visible in the Pacific. **(C)** Same but for LaMa random. The spatial extent of the El Niño is clearly visible. **(D)** Reconstructed temperatures via PConv (*15*). **(E)** Reconstructed temperatures via kriging (*6*). The method fails to reconstruct the spatial extent of the El Niño. **(F)** Temperature anomalies taken from 20CRv3.SI reanalysis. (*8*)

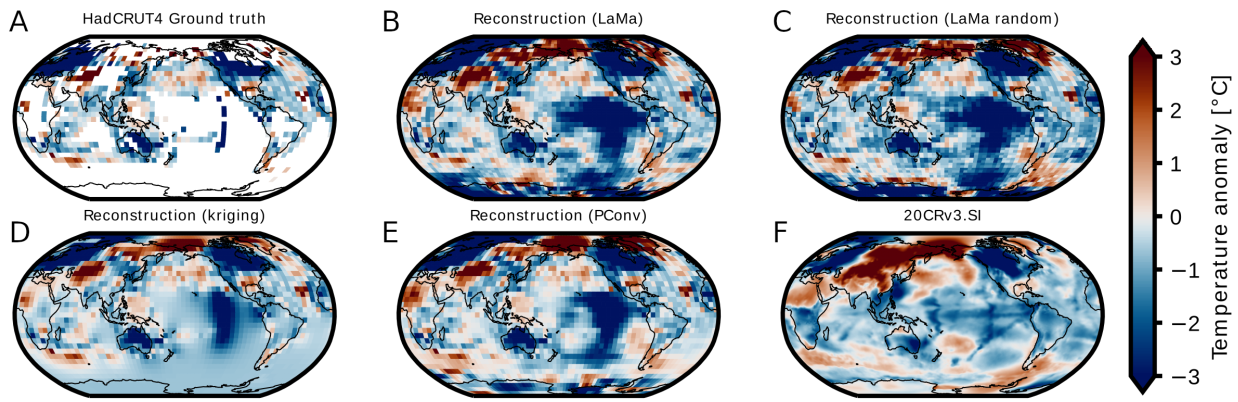

Figure S8: **Reconstructed HadCRUT4 temperature fields for February 1917. (A)** Original HadCRUT4 temperature anomaly records. This also corresponds to the input into the trained model. **(B)** Reconstructed temperature anomalies with LaMa. The strong La Niña is clearly visible in the Pacific. **(C)** Same but for LaMa random. The spatial extent of the La Niña is clearly visible. **(D)** Reconstructed temperatures via PConv (*15*). **(E)** Reconstructed temperatures via kriging (*6*). The method fails to reconstruct the spatial extent of the La Niña. **(F)** Temperature anomalies taken from 20CRv3.SI reanalysis (*8*).

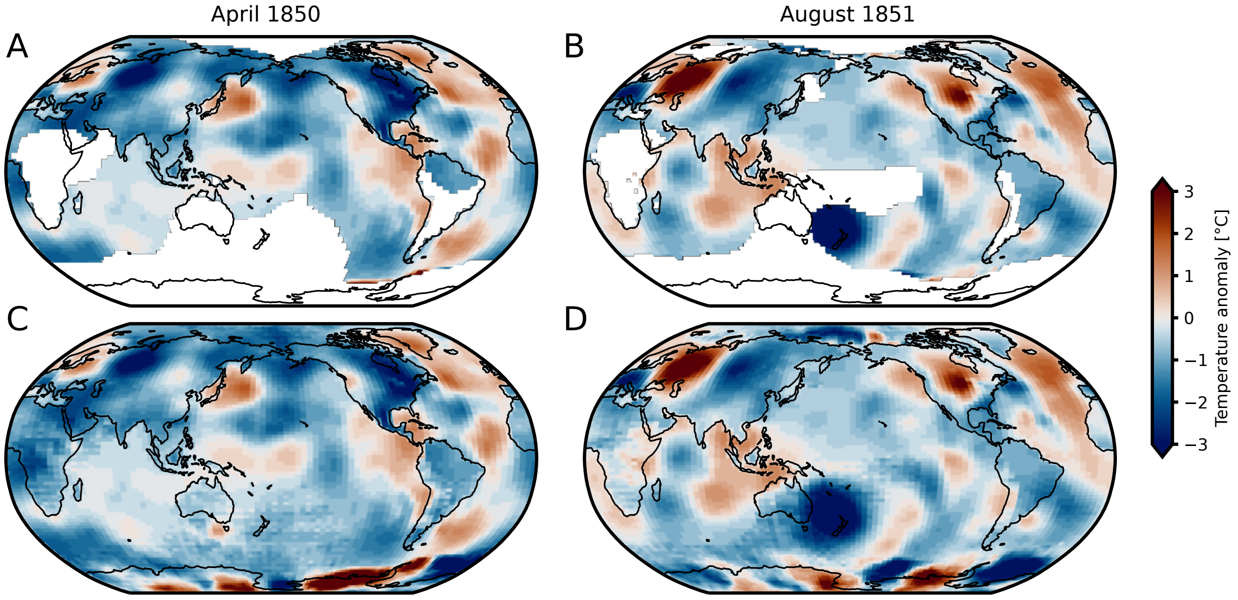

Figure S9: **Reconstruction of two example months for higher resolution BEST data set. (A)** Not inpainted temperature records for April 1850 (BEST dataset, (*42*)). **(B)** Same as **A** but for August 1851. **(C)** Reconstruction of BEST (90x90 px resolution) for April 1850 using LaMa random trained on CMIP5 (72x72 px resolution). **(D)** Same as **(C)** but for August 1851.

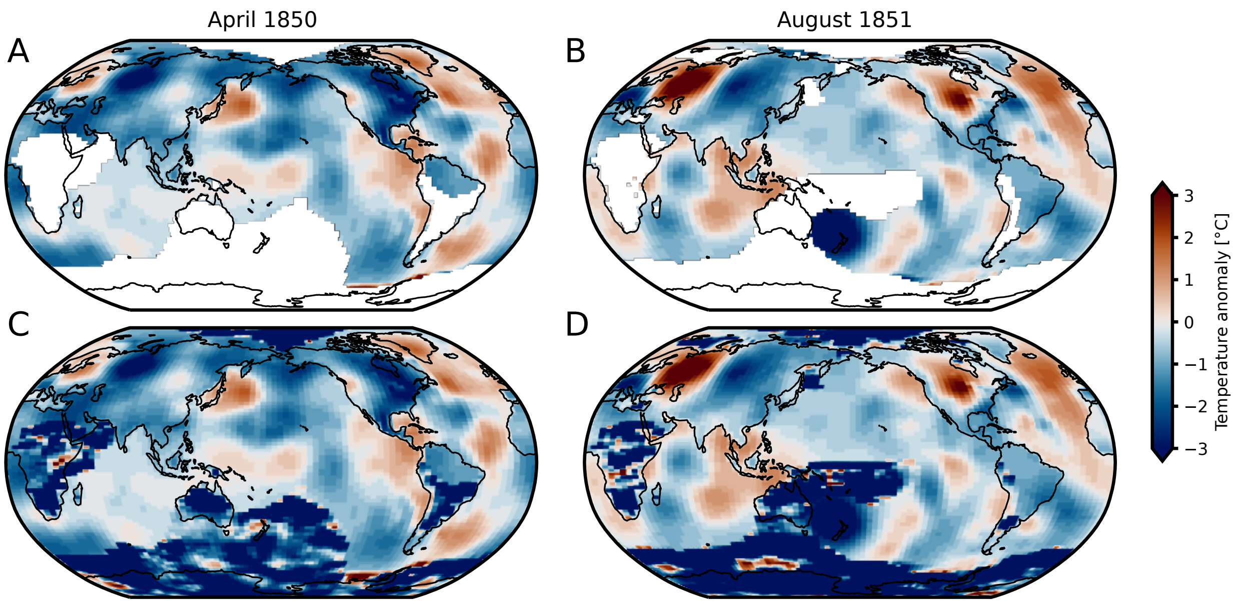

Figure S10: **Example of artifacts of reconstruction for higher resolution BEST data set using LaMa.** **(A)** Not inpainted temperature records for April 1850 (BEST dataset, (*42*)). **(B)** Same as **A** but for August 1851. **(C)** Reconstruction of BEST (90x90 px resolution) for April 1850 using LaMa trained on CMIP5 (72x72 px resolution). Unseen masks lead to obvious artifacts when filled in with LaMa. **(D)** Same as **(C)** but for August 1851.

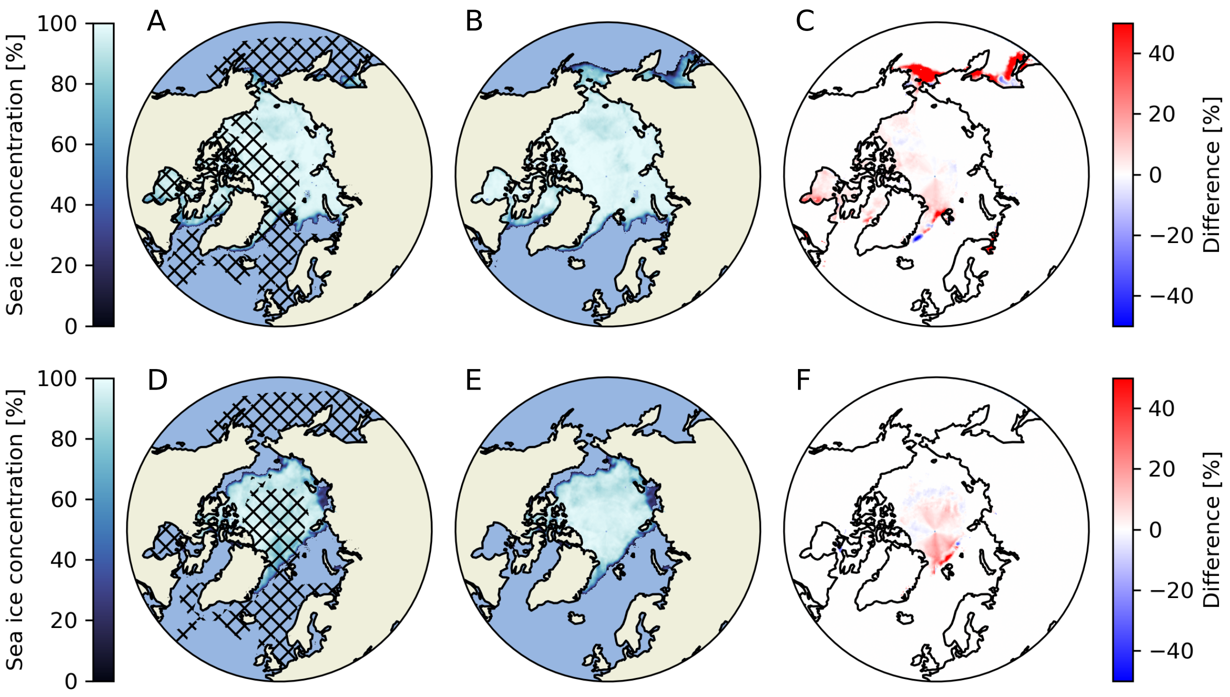

Figure S11: **Example reconstruction of sea ice concentration for two different months in ERA5. (A)** Ground truth of sea ice concentration for held-out December 14, 1979 taken from ERA5 reanalysis (*43*). Hatched area denote regions that are masked for reconstruction. We use LaMa fixed trained on daily sea ice concentration from 1979 to 2022 taken from ERA5 for the reconstruction. **(B)** Reconstructed sea ice concentration via LaMa fixed. The model is able to reconstruct the spatial extent and concentration of the sea ice reasonably well. **(C)** Absolute difference between the ground truth and the reconstructed sea ice concentration. Red areas denote overestimated sea ice concentration by the reconstruction, while blue regions denote underestimated sea ice concentration. **(D,E,F)** Same as **A**, **B**, **C** but for September 16, 1979, respectively.

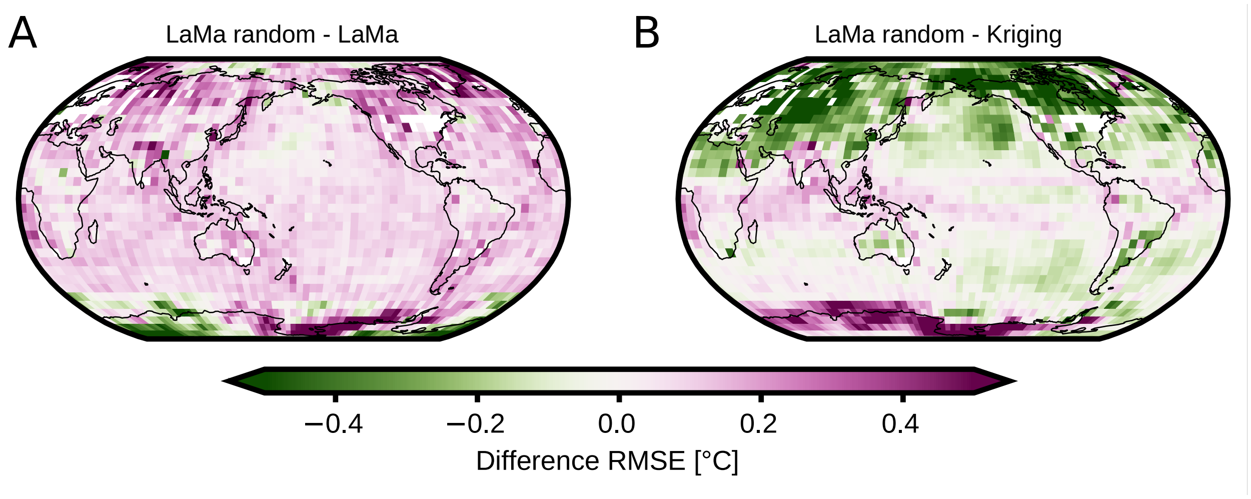

Figure S12: **Difference in site-wise RMSE between LaMa random and LaMa/kriging. (A)** Difference between temporally averaged RMSE at each site between LaMa random and LaMa for held-out CMIP5 member. The white areas denote the regions with available temperature records for the whole time span 1870-2005 AD. Purple areas denote regions where the RMSE of LaMa random is greater than of LaMa. Green areas denote where the RMSE of LaMa random is smaller than for LaMa. LaMa shows a lower RMSE than LaMa random in 82% of the grid cells. **(B)** Same as **A** but for the difference between LaMa random and kriging. LaMa random shows a lower site-wise RMSE than kriging in 65% of the grid cells (green areas).

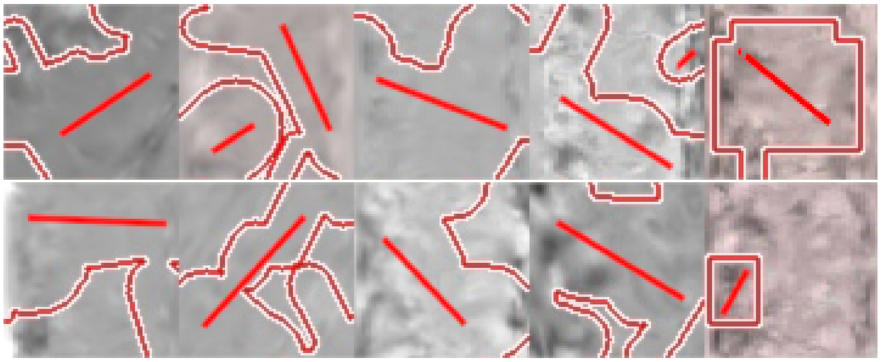

Figure S13: **Example random masks generated during training for LaMa random.** Random masks generated during the training procedure of LaMa random. The masked area is denoted by the red outline and red lines. For details on the generation process see Method section.

REFERENCES AND NOTES

1. M. Ben-Yami, V. Skiba, S. Bathiany, N. Boers, Uncertainties in critical slowing down indicators of observation-based fingerprints of the Atlantic Overturning Circulation. *Nat. Commun.* **14**, 8344 (2023).

2. C. P. Morice, J. J. Kennedy, N. A. Rayner, P. D. Jones, Quantifying uncertainties in global and regional temperature change using an ensemble of observational estimates: The HadCRUT4 data set. *J. Geophys. Res. Atmos.* **117**, D08101 (2012).

3. I. Harris, T. J. Osborn, P. Jones, D. Lister, Version 4 of the CRU TS monthly high-resolution gridded multivariate climate dataset. *Sci. Data* **7**, 109 (2020).

4. E. Vermote, NOAA CDR Program, NOAA climate data record (CDR) of AVHRR normalized difference vegetation index, version 5 (NOAA National Centers for Environmental Information, 2018); www.ncei.noaa.gov/metadata/geoportal/rest/metadata/item/gov.noaa.ncdc:C01558/html.

5. J. C. Landy, G. J. Dawson, M. Tsamados, M. Bushuk, J. C. Stroeve, S. E. L. Howell, T. Krumpen, D. G. Babb, A. S. Komarov, H. D. B. S. Heorton, H. J. Belter, Y. Aksenov, A year-round satellite sea-ice thickness record from CryoSat-2. *Nature* **609**, 517–522 (2022).

6. K. Cowtan, R. G. Way, Coverage bias in the HadCRUT4 temperature series and its impact on recent temperature trends. *Q. J. Roy. Meteorol. Soc.* **140**, 1935–1944 (2014).

7. E. Kalnay, M. Kanamitsu, R. Kistler, W. Collins, D. Deaven, L. Gandin, M. Iredell, S. Saha, G. White, J. Woollen, Y. Zhu, A. Leetmaa, R. Reynolds, M. Chelliah, W. Ebisuzaki, W. Higgins, J. Janowiak, K. C. Mo, C. Ropelewski, J. Wang, R. Jenne, D. Joseph, The NCEP/NCAR 40-year reanalysis project. *Bull. Am. Meteorol. Soc.* **77**, 437–471 (1996).

8. G. P. Compo, J. S. Whitaker, P. D. Sardeshmukh, N. Matsui, R. J. Allan, X. Yin, B. E. Gleason, R. S. Vose, G. Rutledge, P. Bessemoulin, S. Brönnimann, M. Brunet, R. I. Crouthamel, A. N. Grant, P. Y. Groisman, P. D. Jones, M. C. Kruk, A. C. Kruger, G. J. Marshall, M. Maugeri, H. Y. Mok, Ø. Nordli, T. F. Ross, R. M. Trigo, X. L. Wang, S. D. Woodruff, S. J. Worley, The twentieth century reanalysis project. *Q. J. Roy. Meteorol. Soc.* **137**, 1–28 (2011).

9. B. Bell, H. Hersbach, A. Simmons, P. Berrisford, P. Dahlgren, A. Horányi, J. Muñoz-Sabater, J. Nicolas, R. Radu, D. Schepers, C. Soci, S. Villaume, J.-R. Bidlot, L. Haimberger, J. Woollen, C. Buontempo, J.-N. Thépaut, The ERA5 global reanalysis: Preliminary extension to 1950. *Q. J. Roy. Meteorol. Soc.* **147**, 4186–4227 (2021).

10. C. Soci, H. Hersbach, A. Simmons, P. Poli, The ERA5 global reanalysis from 1940 to 2022. *Q. J. Roy. Meteorol. Soc.* **150**, 4014–4048 (2024).

11. L. C. Slivinski, G. P. Compo, P. D. Sardeshmukh, J. S. Whitaker, C. McColl, R. J. Allan, P. Brohan, X. Yin, C. A. Smith, L. J. Spencer, R. S. Vose, M. Rohrer, R. P. Conroy, D. C. Schuster, J. J. Kennedy, L. Ashcroft, S. Brönnimann, M. Brunet, D. Camuffo, R. Cornes, T. A. Cram, F. Domínguez-Castro, J. E. Freeman, J. Gergis, E. Hawkins, P. D. Jones, H. Kubota, T. C. Lee, A. M. Lorrey, J. Luterbacher, C. J. Mock, R. K. Przybylak, C. Pudmenzky, V. C. Slonosky, B. Tinz, B. Trewin, X. L. Wang, C. Wilkinson, K. Wood, P. Wyszyński, An evaluation of the performance of the twentieth century reanalysis version 3. *J. Clim.* **34**, 1417–1438 (2021).

12. T. Berezowski, M. Szcześniak, I. Kardel, R. Michałowski, T. Okruszko, A. Mezghani, M. Piniewski, CPLFD-GDPT5: High-resolution gridded daily precipitation and temperature data set for two largest Polish river basins. *Earth Syst. Sci. Data* **8**, 127–139 (2016).

13. A. Sekulić, M. Kilibarda, D. Protić, M. P. Tadić, B. Bajat, Spatio-temporal regression kriging model of mean daily temperature for Croatia. *Theor. Appl. Climatol.* **140**, 101–114 (2020).

14. L. Belkhiri, A. Tiri, L. Mouni, Spatial distribution of the groundwater quality using kriging and Co-kriging interpolations. *Groundw. Sustain. Dev.* **11**, 100473 (2020).

15. C. Kadow, D. M. Hall, U. Ulbrich, Artificial intelligence reconstructs missing climate information. *Nat. Geosci.* **13**, 408–413 (2020).

16. C. Irrgang, N. Boers, M. Sonnewald, E. A. Barnes, C. Kadow, J. Staneva, J. Saynisch-Wagner, Towards neural Earth system modelling by integrating artificial intelligence in Earth system science. *Nat. Mach. Intell.* **3**, 667–674 (2021).

17. T. Mitsui, N. Boers, Seasonal prediction of Indian summer monsoon onset with echo state networks. *Environ. Res. Lett.* **16**, 074024 (2021).

18. B. Lim, S. Zohren, Time-series forecasting with deep learning: A survey. *Philos. Trans. R. Soc. A* **379**, 20200209 (2021).

19. R. Lam, A. Sanchez-Gonzalez, Matthew Willson, P. Wirnsberger, M. Fortunato, F. Alet, S. Ravuri, T. Ewalds, Z. Eaton-Rosen, W. Hu, A. Merose, S. Hoyer, G. Holland, O. Vinyals, J. Stott, A. Pritzel, S. Mohamed, P. Battaglia, Learning skillful medium-range global weather forecasting. *Science* **382**, eadi2336 (2023).

20. D. Grattarola, P. Vandergheynst, Generalised implicit neural representations. arXiv:2205.15674 [cs, eess] (2022).

21. P. Hess, M. Drüke, S. Petri, F. M. Strnad, N. Boers, Physically constrained generative adversarial networks for improving precipitation fields from Earth system models. *Nat. Mach. Intell.* **4**, 828–839 (2022).

22. Y. Huang, L. Yang, Z. Fu, Reconstructing coupled time series in climate systems using three kinds of machine-learning methods. *Earth Syst. Dyn.* **11**, 835–853 (2020).

23. C. Monteleoni, G. A. Schmidt, S. McQuade, Climate informatics: Accelerating discovering in climate science with machine learning. *Comput. Sci. Eng.* **15**, 32–40 (2013).

24. M. Reichstein, G. Camps-Valls, B. Stevens, M. Jung, J. Denzler, N. Carvalhais, Deep learning and process understanding for data-driven Earth system science. *Nature* **566**, 195–204 (2019).

25. J. Yuval, P. A. O’Gorman, C. N. Hill, Use of neural networks for stable, accurate and physically consistent parameterization of subgrid atmospheric processes with good performance at reduced precision. *Geophys. Res. Lett.* **48**, e2020GL091363 (2021).

26. Y. Zhu, R. H. Zhang, J. N. Moum, F. Wang, X. Li, D. Li, Physics-informed deep-learning parameterization of ocean vertical mixing improves climate simulations. *Natl. Sci. Rev.* **9**, nwac044 (2022).

27. M. Gelbrecht, A. White, S. Bathiany, N. Boers, Differentiable programming for Earth system modeling *Geosci. Model Dev.* **16**, 3123–3135 (2023).

28. T. Schneider, S. Behera, G. Boccaletti, C. Deser, K. Emanuel, R. Ferrari, L. R. Leung, N. Lin, T. Müller, A. Navarra, O. Ndiaye, A. Stuart, J. Tribbia, T. Yamagata, Harnessing AI and computing to advance climate modelling and prediction. *Nat. Clim. Change* **13**, 887–889 (2023).

29. C. O. de Burgh-Day, T. Leeuwenburg, Machine learning for numerical weather and climate modelling: A review. *Geosci. Model Dev.* **16**, 6433–6477 (2023).

30. D. Kochkov, J. Yuval, I. Langmore, P. Norgaard, J. Smith, G. Mooers, M. Klöwer, J. Lottes, S. Rasp, P. Düben, S. Hatfield, P. Battaglia, A. Sanchez-Gonzalez, M. Willson, M. P. Brenner, S. Hoyer, Neural general circulation models for weather and climate. *Nature* **632**, 1060–1066 (2024).

31. Z. Qin, Q. Zeng, Y. Zong, F. Xu, Image inpainting based on deep learning: A review. *Displays* **69**, 102028 (2021).

32. X. Zhang, X. Wang, C. Shi, Z. Yan, X. Li, B. Kong, S. Lyu, B. Zhu, J. Lv, Y. Yin, Q. Song, X. Wu, I. Mumtaz, DE-GAN: Domain embedded GAN for high quality face image inpainting. *Pattern Recognit.* **124**, 108415 (2022).

33. Y. Chen, R. Xia, K. Zou, K. Yang, RNON: Image inpainting via repair network and optimization network. *Int. J. Mach. Learn Cybern.* **14**, 2945–2961 (2023).

34. R. Suvorov, E. Logacheva, A. Mashikhin, A. Remizova, A. Ashukha, A. Silvestrov, N. Kong, H. Goka, K. Park, V. Lempitsky, Resolution-robust large mask inpainting with Fourier convolutions. arXiv:2109.07161 [cs, eess] (2021).

35. D. E. Parker, T. P. Legg, C. K. Folland, A new daily central England temperature series, 1772–1991. *Int. J. Climatol.* **12**, 317–342 (1992).

36. M. Davis, *Late Victorian Holocausts: El Niño Famines and the Making of the Third World* (Verso Books, 2002).

37. B. Huang, M. L'Heureux, Z.-Z. Hu, X. Yin, H.-M. Zhang, How significant was the 1877/78 El Niño? *J. Clim.* **33**, 4853–4869 (2020).

38. B. S. Giese, S. Ray, El Niño variability in simple ocean data assimilation (SODA), 1871–2008. *J. Geophys. Res.* **116**, C02024 (2011).

39. E. N. Voskresenskaya, O. V. Marchukova, Qualitative classification of the La Niña events. *Phys. Oceanogr.* 14–24 (2015).

40. L. C. Slivinski, G. P. Compo, J. S. Whitaker, P. D. Sardeshmukh, B. S. Giese, C. McColl, R. Allan, X. Yin, R. Vose, H. Titchner, J. Kennedy, L. J. Spencer, L. Ashcroft, S. Brönnimann, M. Brunet, D. Camuffo, R. Cornes, T. A. Cram, R. Crouthamel, F. Domínguez-Castro, J. E. Freeman, J. Gergis, E. Hawkins, P. D. Jones, S. Jourdain, A. Kaplan, H. Kubota, F. Le Blancq, T.-C. Lee, A. Lorrey, J. Luterbacher, M. Maugeri, C. J. Mock, G. W. K. Moore, R. Przybylak, C. Pudmenzky, C. Reason, V. C. Slonosky, C. A. Smith, B. Tinz, B. Trewin, M. A. Valente, X. L. Wang, C. Wilkinson, K. Wood, P. Wyszyński, Towards a more reliable historical reanalysis: Improvements for version 3 of the twentieth century reanalysis system. *Q. J. Roy. Meteorol. Soc.* **145**, 2876–2908 (2019).

41. C. P. Morice, J. J. Kennedy, N. A. Rayner, J. P. Winn, E. Hogan, R. E. Killick, R. J. H. Dunn, T. J. Osborn, P. D. Jones, I. R. Simpson, An updated assessment of near-surface temperature change from 1850: The HadCRUT5 data set. *J. Geophys. Res. Atmos.* **126**, e2019JD032361 (2021).

42. R. A. Rohde, Z. Hausfather, The berkeley earth land/ocean temperature record. *Earth Syst. Sci. Data.* **12**, 3469–3479 (2020).

43. Copernicus Climate Change Service (C3S), Sea ice concentration daily gridded data from 1979 to present derived from satellite observations (2020); https://cds.climate.copernicus.eu/cdsapp#!/dataset/satellite-sea-ice-concentration?tab=overview.

44. J. M. Beckers, M. Rixen, EOF calculations and data filling from incomplete oceanographic datasets. *J. Atmos. Oceanic Tech.* **20**, 1839–1856 (2003).

45. K. Wang, G. D. Clow, Reconstructed global monthly land air temperature dataset (1880–2017). *Geosci. Data J.* **7**, 4–12 (2020).

46. T. M. Smith, R. W. Reynolds, R. E. Livezey, D. C. Stokes, Reconstruction of historical sea surface temperatures using empirical orthogonal functions. *J. Climate* **9**, 1403–1420 (1996).

47. M. S. Y. Tang, S. N. Chenoli, A. A. Samah, O. S. Hai, An assessment of historical Antarctic precipitation and temperature trend using CMIP5 models and reanalysis datasets. *Polar Sci.* **15**, 1–12 (2018).

48. R. Keisler, Forecasting global weather with graph neural networks. arXiv:2202.07575 [physics] (2022)

49. S. Scher, G. Messori, Physics-inspired adaptions to low-parameter neural network weather forecasts systems. arXiv:2008.13524 [physics] (2023).

50. C. Esteves, J.-J. Slotine, A. Makadia, "Scaling spherical CNNs," in *Proceedings of the 40th International Conference on Machine Learning* (MLResearchPress, 2023), vol. 202, pp. 9396–9411.

51. A. Lugmayr, M. Danelljan, A. Romero, F. Yu, R. Timofte, L. Van Gool, RePaint: Inpainting using denoising diffusion probabilistic models. arXiv:2201.09865 [cs] (2022)

52. C. Wei, K. Mangalam, P.-Y. Huang, Y. Li, H. Fan, H. Xu, C. Xie, A. Yuille, C. Feichtenhofer, Diffusion models as masked autoencoders. arXiv:2304.03283 [cs] (2023).

53. T. Höppe, A. Mehrjou, S. Bauer, D. Nielsen, A. Dittadi, Diffusion models for video prediction and infilling. arXiv:2206.07696 [cs, stat] (2022).

54. Y. Zeng, J. Fu, H. Chao, B. Guo, Aggregated contextual transformations for high-resolution image inpainting. *IEEE Trans. Vis. Comput. Graph* **29**, 3266–3280 (2021).

55. H. Hukkelås, F. Lindseth, R. Mester, Image inpainting with learnable feature imputation. arXiv:2011.01077 [cs, eess] (2020).

56. R. Rombach, A. Blattmann, D. Lorenz, P. Esser, B. Ommer, High-resolution image synthesis with latent diffusion models. arXiv:2112.10752 [cs] (2022).

57. P. Kulshreshtha, B. Pugh, S. Jiddi, Feature refinement to improve high resolution image inpainting. arXiv:2206.13644 [cs, eess] (2022).

58. L. Chi, B. Jiang, Y. Mu, “Fast fourier convolution,” in *Advances in Neural Information Processing Systems*, H. Larochelle, M. Ranzato, R. Hadsell, M. Balcan, H. Lin, Eds. (Curran Associates, Inc., 2020), vol. 33, pp. 4479–4488.

59. Y. Kilcher, Resolution-robust large mask inpainting with Fourier convolutions (w/ author interview) (2021); www.youtube.com/watch?v=Lg97gWXsiQ4.

60. F. Crameri, G. E. Shephard, P. J. Heron, The misuse of colour in science communication. *Nat. Commun.* **11**, 5444 (2020).

61. B. Murphy, R. Yurchak, S. Müller, GeoStat-Framework/PyKrige: v1.7.0 (2022); https://zenodo.org/record/7008206.

62. Z. Wang, A. C. Bovik, Mean squared error: Love it or leave it? A new look at Signal FidelityMeasures. *IEEE Signal Process. Mag.* **26**, 98–117 (2009). https://ieeexplore.ieee.org/document/4775883.
